# Immunopathological mechanisms in autoimmune encephalitis: from pathogenesis to targeted therapies

**DOI:** 10.3389/fimmu.2026.1837836

**Published:** 2026-09-01

**Authors:** Ying-ying Lu, Shan-chao Zhang, Hai-yun Li, Qi-qing Yin, Yu-qing Fang, Peng Zhou, Shan Qiao, Tao Xin

**Affiliations:** 1 Shandong First Medical University, Jinan, China; 2 Department of Neurology, The First Affiliated Hospital of Shandong First Medical University & Shandong Provincial Qianfoshan Hospital, Jinan, China; 3 Department of Neurology, Beijing Friendship Hospital, Capital Medical University, Beijing, China; 4 Qilu Hospital of Shandong University, Jinan, China; 5 Department of Neurosurgery, The First Affiliated Hospital of Shandong First Medical University & Shandong Provincial Qianfoshan Hospital, Jinan, China; 6 Shandong University of Traditional Chinese Medicine, Jinan, Shandong, China; 7 First Clinical Medical College, Shandong University of Traditional Chinese Medicine, Jinan, Shandong, China; 8 Shandong Engineering Research Center of Precision Diagnosis and Treatment Technology for Neuro-oncology, Jinan, China; 9 Laboratory of Basic and Translational Neuromedicine, The First Affiliated Hospitalof Shandong First Medical University, Jinan, China; 10 Shandong Institute of Brain Scienceand Brain-inspired Research, Jinan, China

**Keywords:** autoimmune encephalitis, immune regulation, immunological diversity, molecular mechanisms, precision medicine

## Abstract

Autoimmune encephalitis (AE) is an antibody-mediated brain disorder involving self-antibody-driven inflammation. Different subtypes present unique clinical and pathological features, influenced by variations in target antigens and underlying pathophysiological processes. Although the precise pathogenesis of each AE subtype remains incompletely understood, recent research has provided valuable insights into their immunopathological mechanisms. This article critically evaluates current discoveries regarding the immunobiology of diverse autoimmune encephalitis categories, elucidating their multifaceted etiological pathways. Furthermore, this review highlights the potential of targeted therapeutic interventions, and personalized treatment strategies, thereby facilitating the translation of mechanism discoveries into precision medicine.

## 1 Introduction

**Figure 1:**
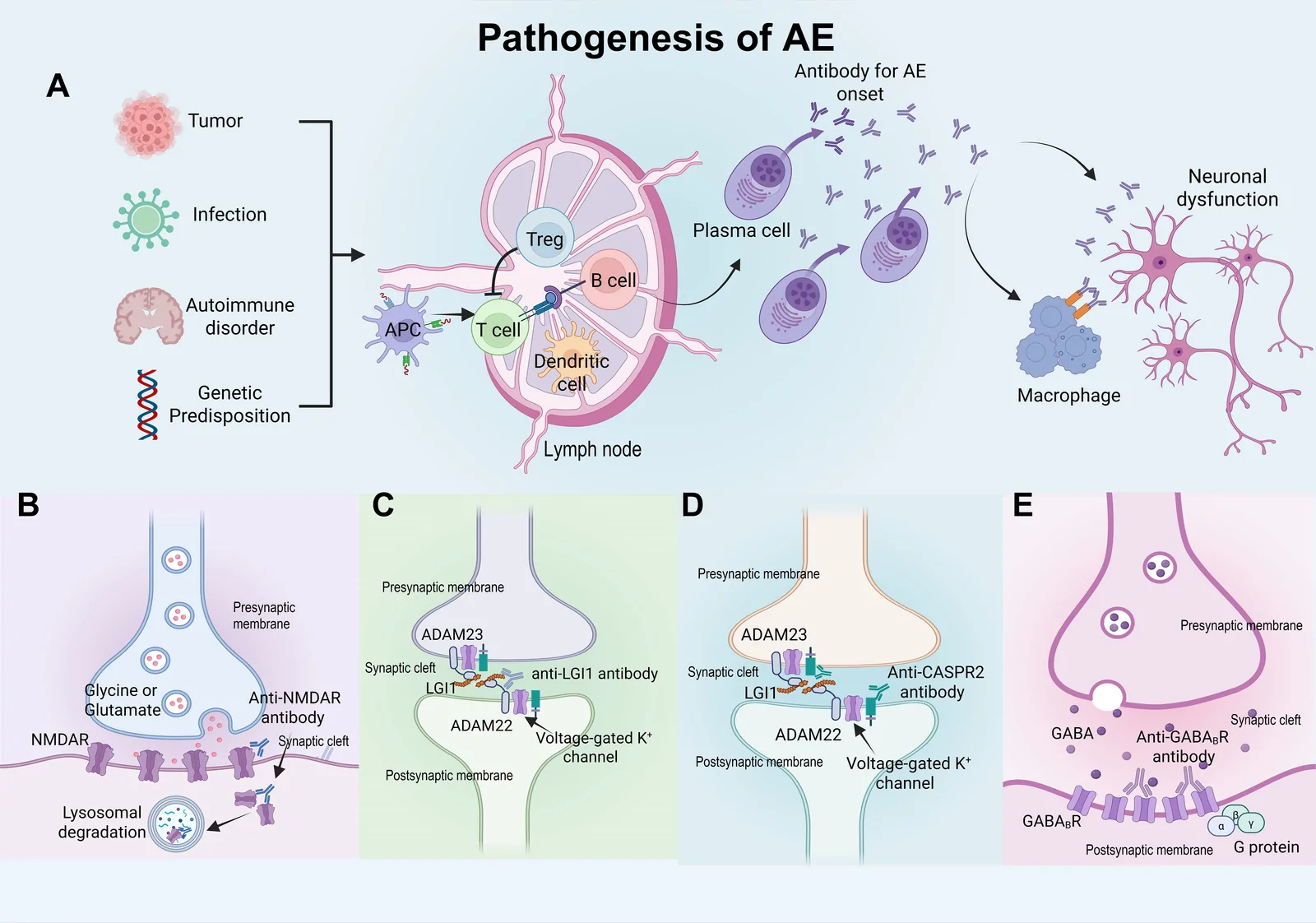
Conceptual schematic of the pathogenesis of AE. **(A)** Multiple triggers—including tumors, infections, autoimmune disorders, and genetic predisposition—initiate immune activation. Antigen-presenting cells (APCs) stimulate T cells, which in turn interact with B cells and plasma cells, leading to the production of pathogenic autoantibodies. These antibodies target neuronal surface or synaptic proteins, and antibody-antigen complexes further recruit macrophages, activate inflammatory cascades, and ultimately induce synaptic injury and neuronal dysfunction, driving the onset of AE. **(B)** Anti-NMDAR encephalitis: antibodies cross-link and internalize NMDAR, reducing surface expression and causing lysosomal degradation, thereby impairing glutamatergic transmission. **(C)** Anti-GABA_B_R encephalitis: antibodies bind to GABA_B_ R, disrupting G protein-coupled inhibitory signaling and leading to excitatory-inhibitory imbalance. **(D)** Anti-LGI1 encephalitis: antibodies block the LGI1-ADAM22/23 complex, destabilizing voltage-gated K+ channel function and synaptic transmission. **(E)** Anti-CASPR2 encephalitis: antibodies interfere with CASPR2 and its interaction with LGI1 and ADAM22/23, also impairing K+ channel regulation. Collectively, these immune-mediated mechanisms converge to produce neuronal hyperexcitability, seizures, and cognitive/psychiatric symptoms typical of AE.

AE comprises a heterogeneous group of neuroinflammatory disorders driven by immune dysregulation (**Figure 1A**). In recent years, multiple antibody-associated subtypes have been identified, including anti-N-methyl-D-aspartate receptor (NMDAR), anti-leucine-rich glioma-inactivated 1 (LGI1), and γ-aminobutyric acid type B receptor (GABA_B_R) encephalitis. These entities differ in clinical presentation, immunopathological mechanisms, and pathological features (1), yet all share a common hallmark of antibody- and/or cell-mediated immune dysfunction leading to neuronal and synaptic injury.

Conceptually, AE can be understood through several interconnected dimensions (**Table 1**). First, the antigenic targets can be broadly classified into neuronal surface or synaptic antigens and intracellular antigens. Antibodies against surface or synaptic proteins, such as NMDAR, LGI1, and GABA_B_R, are more often directly pathogenic by altering receptor trafficking, synaptic transmission, or neuronal excitability. In contrast, antibodies against intracellular antigens are usually considered markers of a broader cytotoxic immune response, in which T-cell-mediated neuronal injury may play a dominant role. Second, the major effector mechanisms vary across subtypes, ranging from IgG-mediated receptor internalization or functional modulation to complement activation and cytotoxic T-cell injury. Third, immune activity may arise from peripheral immune activation, intrathecal antibody synthesis, or both, which has important implications for disease persistence and relapse. Finally, these mechanistic distinctions have direct translational relevance: antibody-removal strategies, B-cell or plasma-cell depletion, cytokine blockade, T-cell-directed therapy, and precision approaches targeting pathogenic antibodies or synaptic receptors may be differentially suited to distinct AE mechanisms.

**Table 1:** Conceptual framework of antigen classes and dominant effector mechanisms in autoimmune encephalitis.

Antigen class	Dominant effector mechanism	Main site of immune activity	Therapeutic implications
Neuronal surface/synaptic antigens, such as NMDAR and LGI1	Mainly IgG-mediated receptor cross-linking, internalization, functional modulation, or disruption of synaptic protein complexes; usually non-cytotoxic and potentially reversible	Immune activation may begin in the periphery, with variable involvement of intrathecal antibody synthesis during disease progression or relapse	Antibody removal and B-cell/plasma-cell-directed therapies are often mechanistically relevant: plasma exchange, IVIG, rituximab, plasma-cell-targeted therapy, FcRn inhibition, and antibody- or receptor-directed precision strategies
Intracellular/onconeural antigens, such as Hu, Yo, and Ma2	Antibodies are usually biomarkers of a broader cytotoxic immune response; neuronal injury is mainly mediated by CD8^+^ T cells and is often less reversible	Often associated with peripheral antitumor immune activation, followed by CNS infiltration of cytotoxic T cells and intrathecal inflammation	Early tumor screening and treatment are critical. T-cell-directed or broader immunosuppressive strategies may be more relevant, including high-dose corticosteroids, cyclophosphamide, and selected small-molecule or cellular immunomodulatory approaches

Although the immune mechanisms and clinical phenotypes of major AE subtypes have been partly clarified, key questions remain regarding how specific antibodies perturb neurotransmitter receptors, immune cell networks, and synaptic function, and how these processes converge or diverge across subtypes. Clinically, earlier diagnosis and improved immunotherapy, including first-line corticosteroids, intravenous immunoglobulin, and plasma exchange, as well as second-line agents such as rituximab and cyclophosphamide, have markedly improved outcomes. However, heterogeneity in treatment response, relapse risk, and therapy-related toxicity underscores the need for more precise and individualized therapeutic strategies guided by antigen class, dominant effector pathway, and the relative contribution of peripheral versus intrathecal immune activity.

This review synthesizes current advances in elucidating the pathogenic basis of AE and summarizes emerging immunotherapeutic and precision medicine approaches. By integrating antigen classification, immune effector mechanisms, sites of immune activity, and therapeutic implications across AE subtypes, it aims to provide a mechanistically organized framework for understanding AE pathophysiology and for informing future research directions and clinical translation.

## 2 Methods

This narrative review searched PubMed and Web of Science for literature published from January 2011 to January 2026. Search terms included combinations of “autoimmune encephalitis”, “antibody-mediated encephalitis”, “anti-NMDAR”, “anti-LGI1”, “anti-CASPR2”, “anti-GABA_B_ receptor”, “anti-DPPX”, “immunopathogenesis”, “B cells”, “T cells”, “cytokines”, “blood-brain barrier”, “HLA”, “immunotherapy”, “targeted therapy” and “precision medicine”. ClinicalTrials.gov was also searched using autoimmune encephalitis-related disease terms and treatment terms such as rituximab, tocilizumab, bortezomib, daratumumab, IVIG, plasma exchange, FcRn inhibitors, and cell therapy.

Original studies, reviews, meta-analyses, guidelines, consensus statements, and relevant case series were included. Duplicates, conference abstracts without full text, and studies unrelated to the immunopathogenesis or treatment of autoimmune encephalitis were excluded. Landmark studies published before 2011 were included when needed for historical context.

When evidence was conflicting, priority was given to human studies, larger cohorts, multicenter studies, systematic reviews, and clinical guidelines. Animal, *in vitro*, small-cohort, and case-report evidence was interpreted cautiously and used mainly to support mechanistic discussion or emerging hypotheses.

Mechanistic evidence was graded into four levels according to the strength of supporting data: Level 1, direct evidence from patient-derived antibody experiments, passive/active animal models, electrophysiology, or human neuropathology; Level 2, probable evidence from consistent cohort, ex vivo, or reproducible biomarker studies; Level 3, associative evidence from cytokine, chemokine, genetic, proteomic, or clinical correlation studies without causal validation; and Level 4, exploratory evidence from case reports, small series, preclinical rationale, or extrapolation from related disorders. This framework was used to distinguish established mechanisms from probable, plausible, and exploratory hypotheses throughout the review.

## 3 Anti-NMDAR encephalitis

**Figure 2:**
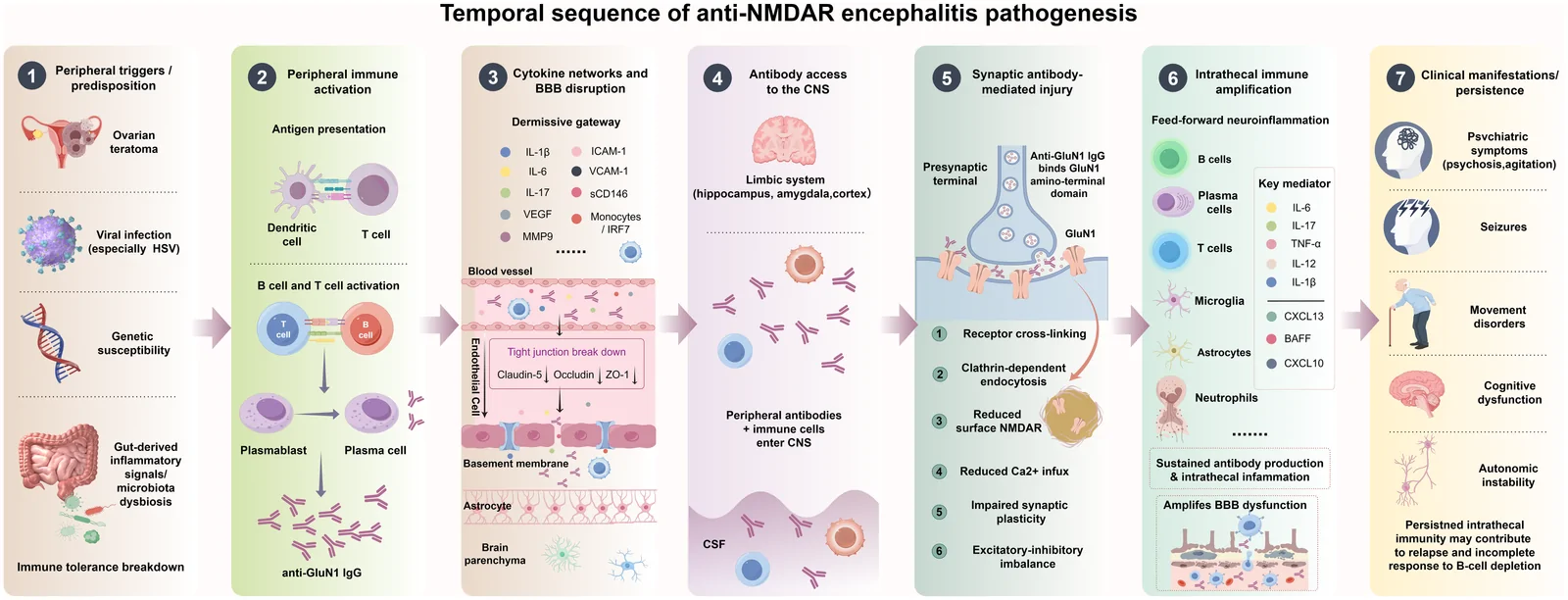
Temporal sequence of anti-NMDAR encephalitis pathogenesis. This conceptual schematic summarizes the stepwise development of anti-NMDAR encephalitis, from peripheral triggers, B- and T-cell activation, and BBB disruption to CNS antibody entry, NMDAR internalization, synaptic dysfunction, intrathecal inflammation, and persistent neurological manifestations.

Anti-NMDAR encephalitis can be understood as a temporally evolving process in which peripheral immune activation, blood-brain barrier (BBB) disruption, antibody entry into the central nervous system (CNS), and intrathecal inflammation interact to drive disease onset and progression. Peripheral triggers, including tumors, viral infection, genetic susceptibility, or gut-derived inflammatory signals, may initiate autoreactive B- and T-cell responses and promote the production of anti-GluN1 antibodies. Cytokine-mediated BBB dysfunction may then provide a permissive gateway for circulating antibodies and immune cells to access the CNS. Once within the brain, anti-GluN1 antibodies induce NMDAR cross-linking, internalization, and synaptic dysfunction, while intrathecal B-cell responses, T-cell-derived cytokines, innate immune mediators, and glial activation may further amplify neuroinflammation. A schematic overview of the proposed pathogenic mechanisms is shown in **Figure 2**.

### 3.1 Disease overview

Anti-NMDAR encephalitis is an immune-mediated CNS disorder caused by autoantibodies against the GluN1 subunit of NMDAR. NMDAR is an ionotropic glutamate receptor composed of GluN1, GluN2(A–D), and GluN3(A–B) subunits, and plays a crucial role in calcium influx, excitatory synaptic transmission, and hippocampal long-term potentiation, which is essential for memory and information storage (2–4). Clinically, the disease often presents with psychiatric symptoms, seizures, movement disorders, autonomic dysfunction, and impaired consciousness (5).

### 3.2 Antibody-mediated mechanisms

Current evidence supports a central pathogenic role for GluN1-specific IgG autoantibodies in anti-NMDAR encephalitis, although the initial events that break immune tolerance remain incompletely defined. Ovarian teratomas and viral infections may trigger autoreactive lymphocyte activation and peripheral production of anti-GluN1 antibodies, which can enter the CNS when BBB integrity is disrupted by inflammation or cytokine-mediated permeability changes.

Within the CNS, anti-GluN1 antibodies bind to the extracellular amino-terminal domain of the GluN1 subunit, with the N368/G369 region identified as an important immunogenic epitope (6). The best-supported mechanism involves antibody-mediated receptor cross-linking and clathrin-dependent endocytosis, leading to reduced surface NMDAR expression at postsynaptic membranes (**Figure 1B**). This receptor loss impairs calcium influx and synaptic transmission, thereby contributing to seizures, cognitive dysfunction, and psychiatric symptoms. Notably, these effects appear reversible, as blocking antibody-receptor interactions or promoting receptor recycling can restore synaptic receptor density, providing a mechanistic basis for immunotherapy and emerging precision strategies such as epitope-specific decoys (7), recombinant ATD-based antibody neutralization, and FcRn inhibition.

EphB2 signaling was previously proposed to stabilize synaptic NMDAR and limit antibody-induced internalization (8). However, recent evidence suggests that disruption of EphB2-NMDAR interactions may not substantially affect receptor distribution, membrane mobility, or electrophysiological function under anti-NMDAR antibody exposure (9). Therefore, in our view, EphB2 may modulate NMDAR function but is unlikely to represent the central driver of antibody-mediated pathology. Passive and active immunization models further demonstrate that NMDAR antibodies are pathogenic and sufficient to induce receptor dysfunction (8, 10). Nevertheless, whether antibodies alone can reproduce the full clinical spectrum of human anti-NMDAR encephalitis remains uncertain, suggesting that additional immune mechanisms may also participate in disease development.

### 3.3 Immune cell-mediated molecular mechanisms

Anti-NMDAR encephalitis is increasingly viewed as an antibody-centered but immune-cell-supported disease (11, 12). Among the established findings, B cells are the direct cellular basis for NR1-IgG production. Human CSF studies show early elevation of CXCL13, supporting B-cell recruitment into the CNS, whereas BAFF is not consistently increased, indicating that B-cell survival is unlikely to be governed by a single cytokine pathway (13–18). B-cell repertoire studies provide further evidence for an antigen-driven humoral response, with NR1-reactive B cells showing restricted BCR usage, shared clonotypes, and clonal expansion in CSF or peripheral blood (19, 20). More recent single-cell multi-omics data suggest expansion of plasma B cells and memory B cells, together with activation of IFNAR-IRF-STAT signaling, which may promote B-cell differentiation and antibody production (21). However, it remains unclear whether pathogenic B-cell responses are mainly initiated in the periphery or locally maintained within the CNS, and which B-cell subsets dominate persistence and relapse.

T cells may support disease development by linking peripheral immune activation with intrathecal antibody responses. CSF studies have reported persistent increases in T-cell-related cytokines, including IFN-γ, TNF-α, IL-17A, and IL-15, while Tcrα-deficient mice lacking mature T cells fail to develop disease after immunization (14, 22). These findings suggest that T cells are unlikely to be passive bystanders. However, the pathogenic T-cell subsets and their precise contribution to B-cell help, CNS inflammation, and disease persistence remain insufficiently defined in human disease.

Innate immune cells and glial responses may further amplify neuroinflammation. Single-cell data suggest activation of monocyte-related TLR2 and TNF signaling pathways during acute disease (21). NETs are increased in patients and correlate with inflammatory cytokines, although whether they directly mediate neuronal injury remains uncertain (23). Cytokines produced by immune and glial cells, including IL-6, IL-17, TNF-α, IL-12, and IL-1β, may further shape the inflammatory cascade (24–26). In our view, innate immune activation and glial inflammation are more likely to function as amplifiers rather than primary initiators of disease. Collectively, pathogenic antibodies remain central, but immune cells determine how the antibody response is initiated, sustained, and amplified.

### 3.4 Molecular mechanisms of BBB disruption

BBB dysfunction is considered a permissive mechanism in anti-NMDAR encephalitis, facilitating the entry of circulating antibodies and immune cells into the CNS. Clinical evidence mainly comes from observational studies showing BBB abnormalities and related biomarkers. Tumor-associated antigen exposure and inflammatory cytokines may contribute to BBB disruption, but the causal link between BBB breakdown and neuropsychiatric symptoms remains unclear (27, 28). DCE-MRI studies further showed increased global BBB permeability in autoimmune encephalitis, even when conventional MRI is normal, and higher baseline cortical or cerebellar permeability may be associated with poorer treatment response, supporting BBB permeability as a potential diagnostic and prognostic biomarker requiring validation (29).

Mechanistic evidence is largely preclinical. Patient-derived sera or antibodies induce behavioral abnormalities in mice with pre-existing BBB compromise, suggesting that BBB disruption facilitates antibody pathogenicity rather than independently causing disease (30, 31). In experimental models, IL-1β disrupts endothelial tight junctions, whereas IL-6 may impair endothelial function through JAK/STAT3-related VEGF and MMP activation and promote Th17-associated inflammation (32–34). Human biomarker studies have reported increased soluble CD146, ICAM-1, VCAM-1, L-selectin, MMP9 and CD44, supporting endothelial activation and leukocyte transmigration, although these findings remain associative (35–38).

Additional pathways remain exploratory. p38 MAPK inhibition preserved BBB integrity and reduced MMP9 expression in NR1 peptide-immunized mice, while IRF7-related monocyte activation has been linked to BBB leakage and tight-junction disruption (39, 40). The microbiota-gut-brain axis may also modulate BBB function, as patient-derived microbiota or microbial products can enhance systemic inflammation, impair BBB integrity and aggravate behavioral phenotypes in experimental models (41–44). In our view, current evidence supports BBB disruption as an important amplifier and gateway for immune entry, but not yet as a proven primary initiator of anti-NMDAR encephalitis. Future studies should distinguish whether BBB injury precedes antibody-mediated synaptic dysfunction or occurs secondary to systemic and CNS inflammation.

### 3.5 Genetic susceptibility

Compared with anti-LGI1 encephalitis, which shows relatively strong and reproducible HLA class II associations, genetic susceptibility in anti-NMDAR encephalitis appears more heterogeneous. Early European studies reported weak or inconsistent HLA signals, suggesting that anti-NMDAR encephalitis is unlikely to be driven by a single dominant HLA allele (45). In contrast, studies in Chinese populations identified significant associations with HLA-DRB1*16:02 and, more recently, HLA-DQB1*05:02, supporting a role for HLA-mediated antigen presentation in disease susceptibility (46–48). Computational peptide-binding analyses provide a plausible mechanistic link between HLA class II molecules and NMDAR-derived antigens, but direct evidence for HLA-restricted autoreactive T-cell activation remains limited.

Beyond the HLA region, GWAS have identified non-HLA loci such as LRRK1, NR1H3, and IFIH1 (48, 49). Among these, IFIH1, which encodes the viral RNA sensor MDA5, provides a biologically plausible link between antiviral immunity and disease initiation, particularly in post-infectious contexts. By contrast, the proposed roles of LRRK1 and NR1H3 in B-cell biology or neuroinflammatory regulation remain largely inferential. More recent KIR and NK-cell-related associations suggest that innate immune surveillance may also modulate susceptibility, but these findings are exploratory and require replication (50).

In our view, current evidence supports a multilayered genetic model rather than a single-gene or single-HLA explanation. HLA class II associations, especially in East Asian cohorts, represent the most consistent genetic evidence, whereas non-HLA loci and KIR/NK-related signals remain emerging findings. Future studies should clarify whether these genetic factors influence disease initiation through antigen presentation, antiviral responses, B-cell activation, or innate immune regulation, and whether population-specific differences reflect ancestry, tumor status, infection history, or disease heterogeneity.

## 4 Voltage-gated potassium channel complex antibody-associated encephalitis

**Figure 3:**
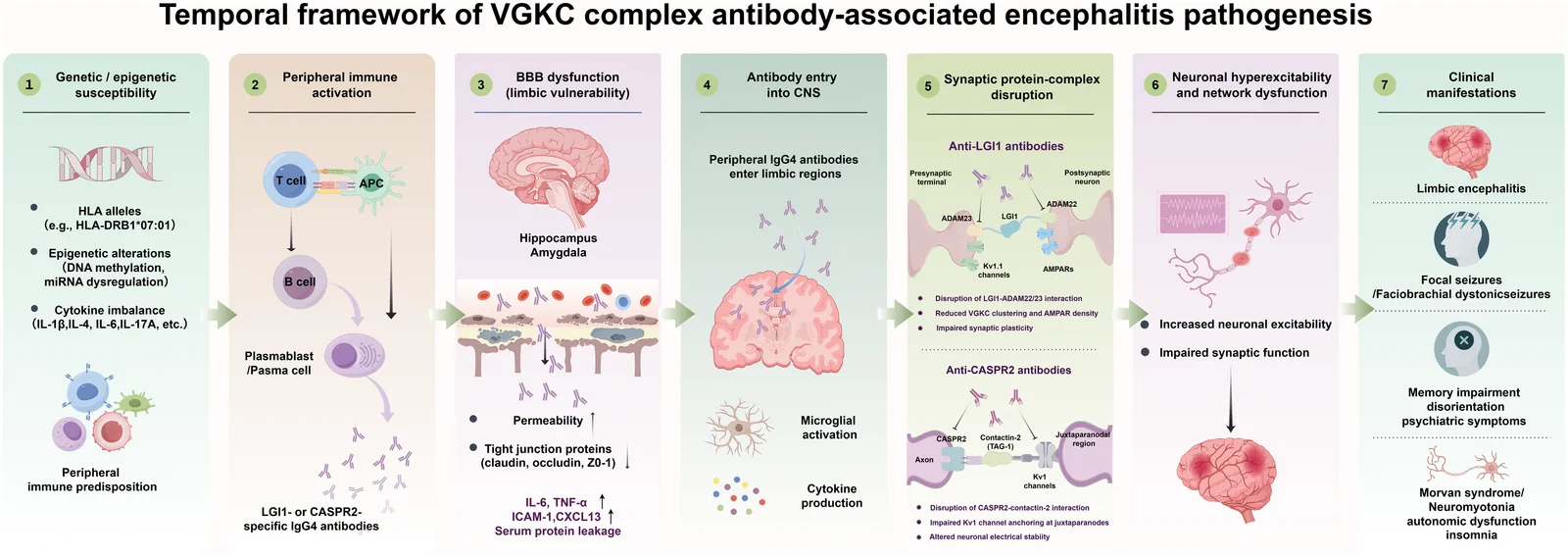
Temporal framework of VGKC complex antibody-associated encephalitis pathogenesis. This conceptual schematic summarizes the proposed pathogenic sequence of VGKC complex antibody-associated encephalitis, particularly anti-LGI1 and anti-CASPR2 encephalitis. Genetic/epigenetic susceptibility and peripheral immune activation promote LGI1- or CASPR2-specific IgG4 antibody production. BBB dysfunction allows antibody entry into limbic regions, where antibodies disrupt LGI1-ADAM22/23 or CASPR2-contactin-2/Kv1 channel complexes, leading to corresponding clinical manifestations.

VGKC complex antibody-associated encephalitis can be understood as a sequential process linking immune predisposition, antibody production, BBB dysfunction, and synaptic disruption. Genetic susceptibility, epigenetic changes, and cytokine imbalance may promote peripheral B- and T-cell activation and the generation of LGI1- or CASPR2-specific IgG4 antibodies. BBB impairment then allows these antibodies to access limbic regions, where they disrupt LGI1-ADAM22/ADAM23 or CASPR2-contactin-2/Kv1 channel complexes, leading to neuronal hyperexcitability and cognitive or seizure-related symptoms. Intrathecal B-cell recruitment, T-cell trafficking, cytokines, and microglial activation may further sustain inflammation and disease activity. A schematic overview of the proposed pathogenic mechanisms is shown in **Figure 3**.

### 4.1 Disease overview

The VGKC complex, including Kv1.1, Kv1.2, Kv1.6 and associated proteins such as LGI1 and CASPR2, is essential for neuronal excitability and synaptic transmission. LGI1 bridges presynaptic ADAM23 and postsynaptic ADAM22 to regulate AMPA receptor-Kv1.1 channel coupling, whereas CASPR2 interacts with contactin-2/TAG-1 and Kv1 channels to stabilize neuronal electrical activity (51–53).

VGKC complex antibody-associated diseases include limbic encephalitis, focal seizures, Morvan syndrome and neuromyotonia. Anti-LGI1 encephalitis typically occurs in older men and presents with faciobrachial dystonic seizures, focal seizures, memory impairment and psychiatric symptoms (54, 55). Anti-CASPR2 encephalitis is characterized by cognitive and neuropsychiatric symptoms, seizures, and sometimes peripheral nerve hyperexcitability or Morvan syndrome (56, 57).

### 4.2 Antibody targeting mechanisms

Anti-LGI1 and anti-CASPR2 encephalitis are predominantly associated with IgG4-subclass antibodies, which have limited ability to activate complement and are thought to mediate disease mainly through functional disruption of neuronal protein interactions rather than cytotoxic injury (58, 59). This IgG4 predominance is a relatively established feature, but why LGI1/CASPR2 autoimmunity preferentially generates IgG4 antibodies, whereas anti-NMDAR encephalitis is mainly IgG1-mediated, remains unresolved. Chronic antigen exposure, T-cell polarization, and cytokine-driven class switching may contribute, but direct evidence is still limited (59–61).

In anti-LGI1 encephalitis, as illustrated in **Figure 1C**, the best-supported mechanism is antibody-mediated disruption of LGI1 interactions with ADAM22 and ADAM23. Patient-derived antibodies recognize the LRR and/or EPTP domains of LGI1, impair LGI1–ADAM binding, and reduce synaptic Kv1.1 clustering and AMPA receptor density in experimental models (62). Domain-specific studies further suggest that LRR-targeting antibodies may promote internalization of LGI1-ADAM complexes, whereas EPTP-targeting antibodies mainly block surface binding; both mechanisms can enhance neuronal excitability, although their relative contribution in patients remains uncertain (63).

Anti-CASPR2 antibodies also appear to act mainly by functional interference. Patient-derived antibodies are largely IgG4 and may recognize extracellular CASPR2 domains, including discoidin and laminin G1 regions, thereby disrupting CASPR2-contactin-2/TAG-1 interactions and Kv1 channel organization without necessarily inducing CASPR2 internalization (64, 65). Recent monoclonal antibody studies provide stronger experimental evidence that selected CASPR2 antibodies can directly alter neuronal function and behavior in rodent models, although the extent to which these mechanisms explain the full human phenotype requires further validation (66). Available data also suggest that antibody-mediated effects may be at least partly reversible after antibody removal or treatment (67).

By contrast, VGKC-complex antibody positivity without LGI1 or CASPR2 reactivity has weaker diagnostic and pathogenic significance. Many such antibodies target intracellular or non-pathogenic epitopes, and VGKC positivity alone does not reliably distinguish autoimmune encephalitis from other conditions; no clear titer threshold has been established for pathogenicity (68, 69). In our view, LGI1- and CASPR2-specific antibodies represent biologically meaningful disease markers with experimentally supported pathogenic mechanisms, whereas isolated VGKC positivity should be interpreted cautiously. Future studies should clarify whether additional clinically relevant VGKC-complex antigenic targets exist beyond LGI1 and CASPR2.

### 4.3 Immune cells and cytokines

Anti-LGI1 and anti-CASPR2 encephalitis involve both peripheral and intrathecal immune responses. In anti-LGI1 encephalitis, frequent CSF antibody detection with uncommon pleocytosis or oligoclonal bands suggests a mainly peripheral antibody response, whereas intrathecal B-cell clonal expansion and somatic hypermutation indicate possible CNS B-cell contribution to antibody maturation or disease persistence (70). In anti-CASPR2 encephalitis, elevated CSF CXCL13 and soluble ICAM-1 suggest B-cell recruitment and T-cell trafficking across the BBB (71). The detection of antigen-specific antibody-secreting cells in the CSF further supports a peripheral-central immune model, in which autoreactive B cells may mature in the periphery before local CNS differentiation (72, 73).

Additional immune pathways may modify disease activity, but their causal roles remain uncertain. CD8^+^ T-cell infiltration and complement deposition have been described in anti-LGI1 encephalitis; however, given the predominance of weakly complement-activating IgG4 antibodies, these findings more likely reflect local immune activation than primary complement-mediated injury (71, 74).

Cytokine and proteomic studies suggest broader inflammatory activation. In anti-LGI1 encephalitis, increased IL-1β, IL-4, IL-6, IL-8/CXCL8 and IL-17A indicate innate immune activation, possible IgG4-favoring polarization, and Th17/myeloid-related inflammation (34, 75, 76). Proteomic data similarly implicate Th1/Th17, myeloid and B-cell-related pathways (77). In anti-CASPR2 encephalitis, increased IL-6, IL-15, BAFF, TARC/CCL17, sCD40L, C5/C5a, BDNF and EGF suggest involvement of innate immunity, adaptive immune activation, complement and neurotrophic signaling (78, 79).

Overall, antibody-mediated neuronal dysfunction remains the best-supported mechanism, whereas B-cell recruitment, T-cell infiltration, complement activation and cytokine changes may amplify or sustain disease activity but still require mechanistic validation.

### 4.4 Genetic associations

Anti-LGI1 encephalitis is strongly associated with HLA-mediated genetic susceptibility. HLA-DRB1*07:01 is the most consistently reported risk allele, and homozygosity may further increase disease risk, suggesting a possible allelic dosage effect (80). Additional associations have been reported for HLA-DRB1*04:02, HLA-DRB1*11:01, HLA-DR7 and DRB4, with some alleles linked to early-onset, more severe or non-paraneoplastic phenotypes (81). In contrast, no clear HLA association has been observed in VGKC intracellular antibody-positive cases (69). However, these findings require validation in larger and ethnically diverse cohorts.

Recent studies further suggest that non-HLA and epigenetic factors may contribute to disease susceptibility. Binks et al. confirmed the HLA-DRB1*07:01 association and identified non-HLA risk loci, including PTPRD, which may act through synaptic or immune-related pathways (82). Altered DNA methylation and increased miR-2467-5p expression in serum exosomes and PBMCs have also been reported (83). This miRNA was linked to reduced PDCD1 and CSF3 expression and increased CCL15 expression, suggesting possible regulation of immune-checkpoint, cytokine and chemokine pathways. Overall, genetic and epigenetic data provide important clues to anti-LGI1 disease susceptibility, but their functional relevance to T/B-cell regulation, inflammatory recruitment and antibody production remains largely inferential.

### 4.5 BBB dysfunction

Recent studies indicate that BBB disruption may play an important contributory role in the pathogenesis of anti-LGI1 encephalitis. BBB impairment appears to be regionally selective in experimental models, predominantly affecting the hippocampus and amygdala, and is characterized by increased vascular permeability, immunoglobulin leakage, and downregulation or disruption of tight junction proteins (84). These structural alterations, together with microglial activation and elevated cytokines such as IL-6 and TNF-α, facilitate peripheral antibody entry into the CNS and could contribute to a vicious cycle of inflammation and barrier damage. Such findings suggest that BBB protection and repair may represent potential adjunctive therapeutic strategies in anti-LGI1 encephalitis, although this possibility remains to be validated in human studies.

## 5 Anti-GABABR encephalitis

**Figure 4:**
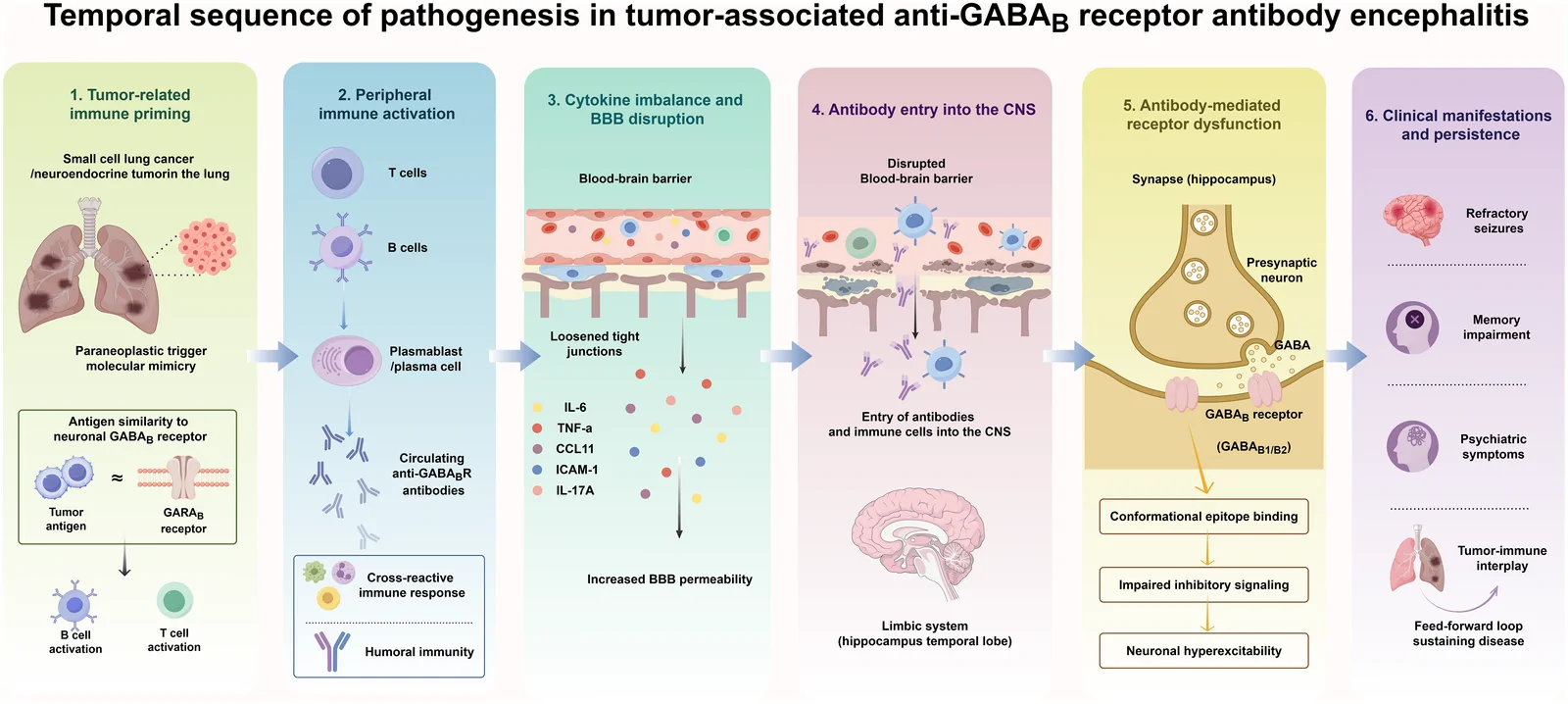
Temporal sequence of pathogenesis in tumor-associated anti-GABA_B_ receptor antibody encephalitis. This conceptual schematic illustrates how tumor-related immune priming, particularly small-cell lung cancer, may trigger cross-reactive B- and T-cell responses, circulating anti-GABA_B_R antibodies, cytokine-mediated BBB disruption, and antibody entry into the CNS. Binding of anti-GABA_B_R antibodies at hippocampal synapses impairs inhibitory signaling, leading to neuronal hyperexcitability and clinical manifestations such as refractory seizures, memory impairment, psychiatric symptoms, and sustained tumor-immune interactions.

Anti-GABA_B_R encephalitis can be viewed as a multistep immune-mediated disorder linking tumor-related immune activation, systemic and intrathecal antibody responses, possible BBB disruption, antibody access to the CNS, and synaptic inhibitory dysfunction. In a substantial proportion of patients, SCLC or other neuroendocrine tumors may ectopically express neuronal antigens or antigenically related proteins, thereby triggering cross-reactive immune responses and promoting the production of GABA_B_R-specific antibodies. However, this tumor-immune-CNS axis should be interpreted as a proposed model rather than a fully established causal sequence. Cytokine/chemokine imbalance and immune-checkpoint dysregulation may further contribute to systemic immune activation and could facilitate neuroinflammation or BBB dysfunction, allowing antibodies and immune cells to enter the limbic system. Within the CNS, anti-GABA_B_R antibodies may interfere with receptor-mediated inhibitory signaling, leading to disrupted excitatory-inhibitory balance, seizures, cognitive impairment, and neuropsychiatric symptoms, while glial activation and inflammatory cytokines may further amplify disease activity. A schematic overview of the proposed pathogenic mechanisms is shown in **Figure 4**.

### 5.1 Disease overview

GABA_B_R is a heterodimer of GABA_B_1 and GABA_B_2, widely expressed in the limbic system. It inhibits glutamate release and regulates synaptic inhibition, and its dysfunction causes neuronal hyperexcitability, seizures, and neuropsychiatric symptoms (85). Encephalitis associated with anti-GABA_B_R antibodies is a rare subtype of AE, initially documented in 2010 (86). The clinical manifestations are primarily characterized by refractory seizures (especially focal seizures originating from the temporal lobe), neuropsychiatric symptoms (such as anxiety and hallucinations), and rapidly progressive memory impairment. Approximately 50% of affected individuals have concomitant small cell lung cancer (SCLC) or other neuroendocrine tumors. However, the reported tumor frequency varies considerably across cohorts, ranging from approximately one-third to more than one-half of patients, with SCLC being the most common associated malignancy.

### 5.2 Paraneoplastic mechanism as the dominant pathogenesis

Several studies have emphasized the strong association between anti-GABA_B_R encephalitis and malignancies, with 30.4% to 71.4% of patients presenting with tumors, predominantly SCLC (87–90). The proposed mechanism is that tumor cells express antigens similar to neuronal GABA_B_R, triggering a cross-immune response, known as the molecular mimicry hypothesis. The co-localization of GABA_B_R with neuroendocrine tumor and SCLC tissues further supports this hypothesis (88). Nevertheless, direct mechanistic evidence remains limited, and the paraneoplastic model may not explain all cases, especially those without detectable tumors. Therefore, repeated tumor screening and long-term surveillance are clinically important, particularly in older patients, smokers, and patients with poor response or relapse.

### 5.3 Antibody-mediated neurological dysfunction

The strong binding of GABA_B_R antibodies to live cultured hippocampal neurons, coupled with the favorable patient response to immunotherapy, supports a pathogenic role for these antibodies (7). The antibodies bind directly to GABA_B_R and may interfere with receptor-mediated inhibitory signaling, thereby contributing to epilepsy, cognitive impairment, and neuropsychiatric symptoms (87, 90, 91) (**Figure 1E**). Preliminary studies suggest that GABA_B_R antibodies target conformational epitopes of the receptor, potentially altering synaptic function rather than reducing receptor levels at the synapse (92). Thus, the pathogenic mechanism is better described as functional disruption of GABA_B_R-mediated synaptic modulation rather than simple receptor loss. Furthermore, the coexistence of other autoantibodies, such as VGCC antibodies and anti-IgLON5 antibodies, has been reported in some patients and may be associated with more complex clinical manifestations (93, 94), suggesting that overlapping antibody responses could contribute to clinical heterogeneity. Other paraneoplastic antibodies, such as anti-Hu and SOX1 antibodies, may also coexist in some tumor-associated cases and could partly explain the heterogeneity of clinical manifestations, tumor association, and prognosis.

### 5.4 Humoral immunity and inflammatory immune responses

Antibodies may enter the CNS through regions with BBB disruption (94, 95). Upon breaching the BBB, GABA_B_R-targeting antibodies may contribute to microglial/astrocytic activation, with subsequent pro-inflammatory cytokine release (e.g., IL-6, TNF-α). This cascade could intensify local inflammation and contribute to neuronal damage. However, direct evidence that anti-GABA_B_R antibodies themselves induce glial activation remains limited; therefore, this process should be interpreted as a plausible but not fully established mechanism. Furthermore, the symptomatic improvement observed with immunotherapies, including corticosteroids, IVIG, plasma exchange and rituximab, provides indirect evidence of the crucial involvement of humoral immunity in disease pathogenesis.

The pathogenesis of anti-GABA_B_R encephalitis is closely linked to dysregulation of cytokine/chemokine networks and abnormalities in immune checkpoint molecules. Rather than acting as isolated inflammatory markers, these cytokine, chemokine, and immune-checkpoint abnormalities may reflect a broader subtype-specific immune network involving both peripheral immune activation and CNS inflammation. Compared to healthy controls, patients with anti-GABA_B_R encephalitis have been reported to show altered serum cytokine and chemokine profiles, including changes in inflammatory, chemotactic, and immune-regulatory molecules (96). These imbalances in cytokines and chemokines suggest an abnormal inflammatory response that may contribute to disease pathogenesis. Further research has revealed that serum levels of soluble ICOS (sICOS) and soluble ICOSL (sICOSL) are elevated, while soluble CD86 (sCD86) levels are reduced in patients with anti-GABA_B_R encephalitis (96). sICOS and sICOSL regulate T-cell activation and differentiation, and their dysregulation may reflect altered immune-checkpoint signaling and could contribute to immune-mediated neuronal damage in this disease. Importantly, these findings should be interpreted as associative biomarker changes rather than direct evidence of causality.

Recent CSF data support an inflammatory component in anti-GABA_B_R encephalitis. Compared with LGI1-associated encephalitis, anti-GABA_B_R encephalitis shows a CSF profile more similar to anti-NMDAR encephalitis, with more frequent abnormalities in leukocyte count, protein, oligoclonal bands, and blood-CSF barrier parameters (76), indicating measurable CNS inflammation.

Serum cytokine analyses suggest that IL-1β, IL-10, IL-4, and MCP-1 may help distinguish AE subtypes and correlate with CSF inflammatory indices, including leukocyte count and CSF/serum albumin ratio (76). In anti-GABA_B_R encephalitis, IL-1β is associated with CSF inflammatory changes, implying a possible link between peripheral inflammation and blood–CSF barrier dysfunction, although causality remains unclear.

Serum proteomics further indicates peripheral immune dysregulation. CSF1R and AZGP1 have been proposed as potential diagnostic markers, along with increased signatures of M1 macrophages and activated NK cells (97). These findings suggest involvement of innate immune responses, but given the small cohort, these markers remain exploratory.

Approximately half of the patients have coexisting tumors, although the reported frequency varies across cohorts. In these patients, inflammatory factors such as IL-1β and TCA-3 may be associated with tumor comorbidity and could influence the interplay between encephalitis and malignancy through pro-tumor inflammation and immune suppression, thereby potentially contributing to disease progression. However, whether these factors directly mediate the interaction between tumors and anti-GABA_B_R encephalitis remains unclear. Future studies integrating tumor tissue analysis, paired serum–CSF antibody profiling, longitudinal cytokine/chemokine detection, immune-cell profiling, and proteomic validation in larger cohorts are needed to clarify how tumor-related immune activation, blood–CSF barrier dysfunction, and CNS inflammation interact during disease initiation and progression.

## 6 DPPX antibody-associated encephalitis

Dipeptidyl-peptidase-like protein-6 (DPPX) is an auxiliary subunit of the voltage-dependent potassium channel Kv4.2, which is widely expressed in the CNS, particularly in the hippocampus and cerebellum. Under physiological conditions, Kv4.2 channels generate the A-type potassium current (IA), which limits repetitive neuronal firing and contributes to the regulation of dendritic excitability and action potential propagation.

Anti-DPPX antibody-associated encephalitis is a rare form of AE. CNS manifestations include seizures, cognitive impairment, and psychiatric disturbances (98), whereas cerebellar involvement may lead to ataxia and nystagmus (99). A striking feature of this disease is frequent profound weight loss and chronic diarrhea (100, 101). These gastrointestinal symptoms, together with cognitive or psychiatric impairment and manifestations of CNS hyperexcitability, constitute a recognizable clinical pattern of anti-DPPX encephalitis (102). Infectious triggers, immune dysregulation, and paraneoplastic processes have been implicated as potential etiological factors (100).

Pathogenically, patient-derived IgG antibodies directly bind to neuronal surface DPPX, the auxiliary subunit of Kv4.2 A-type potassium channels. Further mechanistic studies showed that anti-DPPX antibodies are mainly IgG1 and IgG4 subclasses, indicating that both antibody-mediated antigen modulation and subclass-related immune mechanisms may participate in disease development (102). In experimental systems, this binding can result in acute hyperexcitability of neurons in both the enteric and CNS, consistent with network overactivation. At the cellular level, patient IgG reduces surface DPPX clusters and is accompanied by decreased membrane Kv4.2 expression; because these antibodies do not directly recognize Kv4.2, Kv4.2 loss is considered a secondary effect of DPPX disruption (102). In the subacute and chronic phases, disruption of the DPPX-Kv4.2 complex may impair membrane localization and stability, leading to selective downregulation of DPPX and Kv4.2 expression on neuronal membranes. Consequently, IA currents are reduced, repolarization is compromised, and sustained neuronal hyperexcitability ensues. Peripherally, this leads to excessive activation of enteric neurons followed by secondary exhaustion, which may account for early persistent diarrhea and weight loss and, in some patients, later hypomotility, whereas centrally it may contribute to tremor, myoclonus, seizures, and cognitive or psychiatric symptoms (103). Importantly, the antibody-induced reduction of DPPX/Kv4.2 surface expression can recover after antibody removal, suggesting that this pathogenic process is at least partly reversible and providing a mechanistic basis for the clinical response to immunotherapy (102).

## 7 GluRδ2 antibody-associated encephalitis

GluRδ2 (also known as GRID2/δ2 receptor) is a member of the glutamate receptor family that is specifically expressed in Purkinje cells of the mammalian cerebellum. It is highly enriched in the postsynaptic density of parallel fiber—Purkinje cell (PF-PC) synapses and serves as a key molecule for maintaining synaptic plasticity and synaptic architecture in the cerebellum (104, 105). Clinically, GluRδ2 antibody-associated encephalitis is characterized predominantly by cerebellar ataxia, often accompanied by dizziness, dysarthria, and gait instability. In some patients, seizures or psychiatric symptoms may also occur, and most cases respond favorably to corticosteroid therapy (106–110).

The etiology and pathogenic role of anti-GluRδ2 autoantibodies remain incompletely defined. In the case reported by Miske et al., the detection of high-titer GluRδ2 antibodies after allogeneic transplantation provided early clinical evidence linking GluRδ2 autoimmunity to an acquired immune-mediated neurological syndrome. More importantly, immunological assays suggested that the antibodies recognized an extracellularly accessible epitope, supporting the possibility that anti-GluRδ2 antibodies could directly interact with neuronal surface GluRδ2 rather than merely representing nonspecific intracellular reactivity (109). However, this evidence came from a single case, and corticosteroid responsiveness only indirectly supports immune involvement; it does not establish the precise pathogenic mechanism. Subsequent work by Berridge et al. further strengthened this concept by showing that patient IgG in pediatric opsoclonus-myoclonus-ataxia syndrome could bind surface-expressed GluD2/GluRδ2 in live cell-based assays. Nevertheless, GluD2 adsorption only partially reduced cerebellar tissue staining, suggesting that GluRδ2 may be an important but not exclusive immune target in these cerebellar autoimmune syndromes (111).

However, compared with better-characterized antibody-mediated encephalitides, the functional pathogenic mechanisms of anti-GluRδ2 antibodies remain insufficiently proven. Future studies should determine whether patient IgG alters GluRδ2 surface expression, disrupts PF-PC synaptic architecture, changes Purkinje cell firing or LTD, and whether these effects are reversible after antibody removal or immunotherapy.

## 8 AE associated with anti-neuronal intracellular antibodies

AE can be categorized into three types based on the site of antigen-antibody interaction: encephalitis linked to anti-neuronal surface antibodies, encephalitis involving antibodies against intracellular synaptic antigens, and encephalitis associated with antibodies against intracellular neuronal antigens. Anti-neuronal intracellular antibody-associated encephalitis includes syndromes related to anti-Hu, anti-Yo, anti-Ri, and anti-CV2 antibodies, which are strongly associated with various malignancies and exhibit poor responses to immunotherapy. The precise mechanisms underlying their pathogenesis remain unclear.

### 8.1 Anti-Hu antibody-associated encephalitis

Limbic encephalitis, peripheral neuropathy, cerebellar dysfunction, autonomic disturbances, seizures, and status epilepticus represent the characteristic clinical manifestations of anti-Hu antibody-associated encephalitis, alternatively termed ANNA-1 encephalitis (112). Anti-Hu antibodies are strongly paraneoplastic and are most frequently associated with malignancies, particularly SCLC.

Anti-Hu antibodies can be internalized by cerebellar and hippocampal neurons and have been shown to induce neuronal apoptosis *in vitro*, suggesting a potential direct pathogenic effect. However, whether anti-Hu antibodies contribute substantially to neuronal injury *in vivo* remains controversial, as accumulating pathological evidence supports a dominant role for cytotoxic T-cell-mediated neuronal damage (113). Tumor cells expressing Hu antigens can trigger an immune response, which mistakenly targets CNS-expressed homologous target proteins. Plonquet et al. conducted a study showing that patients with anti-Hu syndrome possess T-cell clones in their bloodstream. T-cell clones not only directly damage sensory neurons but may also inhibit tumor growth via immune-mediated mechanisms, the presence of Hu-specific T-cell clones and the frequent detection of CD8^+^ T-cell infiltrates in affected tissues support the current view that cellular immunity is a major driver of neuronal injury in anti-Hu-associated encephalitis, although the relative contribution of antibodies remains unresolved (114). Consistently, recent tumor transcriptomic data showed that anti-Hu-associated SCLC is enriched for T-cell and IFN-γ-related immune signatures, without consistent mutations or differential expression of ELAVL1–4, suggesting that immune tolerance breakdown is more likely driven by tumor immune context than by structural alteration of Hu antigens themselves (115). At the antigenic level, high-resolution epitope mapping identified a restricted immunodominant motif shared by neuronal ELAVL2/3/4 proteins, overlapping with previously reported Hu-specific T-cell antigenic regions; this finding links humoral anti-Hu reactivity with T-cell antigen recognition (116). Host genetic background may further modulate this response, as HLA-DR3~DQ2 was associated with anti-Hu PNS, particularly in cancer-associated sensory neuropathy, but not in pure CNS involvement, indicating immunopathogenic heterogeneity across anti-Hu phenotypes (117).

In our view, these converging lines of evidence support a working hypothesis that anti-Hu-associated encephalitis primarily reflects a tumor-driven breakdown of immune tolerance, in which restricted neuronal ELAVL antigen recognition and T-cell-dominant neuroinflammation play central roles, whereas the direct pathogenic contribution of anti-Hu antibodies remains uncertain and likely secondary.

### 8.2 Anti-Yo antibody-associated encephalitis

Classified as anti-Purkinje cell cytoplasmic antibody type 1 (PCA-1), the IgG autoantibodies anti-Yo specifically bind cytoplasmic components of Purkinje neurons. Anti-Yo antibodies are predominantly associated with paraneoplastic cerebellar degeneration (PCD), a major subtype of autoimmune cerebellar ataxia, primarily affecting women around 60 years of age with gynecological malignancies, such as ovarian and breast cancer. The hallmark clinical feature is cerebellar ataxia (118).

Initially, cerebellar degeneration-related protein 2 (CDR2), a 62-kDa protein normally expressed in cerebellar tissue but aberrantly found in tumors, was considered the main antigen recognized by anti-Yo antibodies. Additionally, studies have reported CDR2 expression in vascular smooth muscle cells of humans and rats, as well as in ependymal cells lining the cerebral ventricles in rat models (119). Recent investigations reveal that the 55-kDa CDR2L protein, concentrated in Purkinje cells and brainstem neurons, constitutes a novel major antigenic target for anti-Yo autoantibodies. While the pathogenic significance is still unclear, the 34-kDa cerebellar antigen CDR34, localized to Purkinje neurons and featuring repetitive sequences with immunogenic epitopes, has been established as an additional target (120).

Current evidence strongly implicates cytotoxic CD8^+^ T lymphocytes in the pathogenesis of anti-Yo-associated PCD; however, the precise contribution of anti-Yo antibodies remains a subject of ongoing debate. Postmortem studies have demonstrated extensive Purkinje cell degeneration, microglial nodules containing granzyme B-positive CD8^+^ T cells, and neuronal upregulation of MHC class I molecules, supporting a T-cell-mediated mechanism of injury (121, 122). Nevertheless, the pathogenic role of anti-Yo antibodies has not been fully clarified. Although *in vitro* studies indicate that anti-Yo antibodies can enter Purkinje cells and induce neuronal death independently of T lymphocytes, comparable evidence demonstrating a direct pathogenic effect *in vivo* remains limited (123, 124). Therefore, whether anti-Yo antibodies act as direct effectors of neuronal injury or primarily serve as markers of an underlying T-cell response remains unresolved.

### 8.3 Anti-Ri antibody-associated encephalitis

Anti-Ri antibodies, also known as ANNA-2, are paraneoplastic IgG autoantibodies that recognize neuronal nuclear antigens. Their major targets are NOVA-1 and NOVA-2, RNA-binding proteins involved in the regulation of alternative splicing of neuronal transcripts, including those encoding synaptic proteins. Unlike Hu/ELAVL antigens, which are expressed in both central and peripheral neurons, Ri/NOVA antigens are mainly expressed in central neurons; this distribution may partly explain why anti-Ri syndromes are usually dominated by CNS manifestations rather than peripheral neuropathy (125). Anti-Ri antibodies show strong clinical correlation with mammary carcinoma and pulmonary small cell neoplasms (126). Clinically, anti-Ri-associated disease may present with limbic, brainstem, cerebellar, or myelitic involvement, consistent with the broad CNS expression of NOVA proteins.

Available pathological evidence suggests that cytotoxic T cells contribute substantially to disease progression; however, the pathogenic role of anti-Ri antibodies is less clear and may differ from that observed in neuronal surface antibody-mediated encephalitis. This interpretation is supported by the broader framework of intracellular antigen-associated encephalitis, in which antibodies are often considered markers of an underlying neuron-directed immune response, whereas neuronal loss is more strongly linked to cytotoxic CD8^+^ T-cell infiltration, microglial activation, gliosis, and neuronophagia (125). Anti-Hu, anti-Ri, or anti-Ma antibodies can induce paraneoplastic brainstem encephalitis, characterized neuropathologically by perivascular B-cell infiltrates and parenchymal cytotoxic T-cell infiltration (127). Postmortem examinations of anti-Ri antibody-positive patients reveal IgG deposition in brain tissue accompanied by severe Purkinje cell loss and gliosis, with perivascular infiltration of CD4^+^ T cells and B cells alongside parenchymal CD8^+^ T cell infiltration observed in brainstem and midbrain regions (128). Thus, IgG deposition and B-cell infiltrates indicate that humoral immunity is present in affected tissue, but they do not by themselves prove that anti-Ri antibodies are the principal mediators of neuronal injury.

Interestingly, experimental studies suggest that anti-Ri antibody-associated neuronal dysfunction may be at least partially reversible. Unlike anti-Hu antibodies, anti-Ri antibodies impaired neuronal viability without causing marked neuronal death, raising the possibility that different intracellular antibody-associated syndromes may involve distinct pathogenic mechanisms (113). In our view, the most cautious interpretation is that anti-Ri disease may contain two mechanistic layers: a dominant paraneoplastic T-cell-mediated process responsible for progressive neuronal injury, and a possible antibody-related functional component that may contribute to neuronal dysfunction in selected contexts. However, direct *in vivo* evidence for a pathogenic effect of anti-Ri antibodies remains limited, and the relative contribution of humoral versus cellular immunity requires further clarification.

### 8.4 Anti-CV2 antibody-associated encephalitis

Autoantibodies directed against the oligodendrocytic cytoplasmic protein collapsin response mediator protein 5 (CRMP5), termed anti-CV2, exhibit specificity for intraneuronal antigens. These antibodies have been linked to limbic encephalitis, cerebellar ataxia, chorea, peripheral and autonomic neuropathy, myelopathy, as well as optic neuritis and retinopathy (129). Malignancies are detected in over 80% of anti-CV2 antibody-positive cases, with SCLC and thymoma representing the most frequent oncological associations. Immune checkpoint inhibitors may also trigger or worsen CV2/CRMP5-related PNS, suggesting that disruption of tumor immune tolerance can amplify this paraneoplastic response (130).

Research has demonstrated that anti-CV2 antibodies predominantly target CRMP5 and CRMP3, with a weaker reactivity toward CRMP1 (131). The neurological deficits associated with anti-CV2/CRMP5 antibodies are generally thought to result predominantly from CD8^+^ cytotoxic T-cell-mediated neuronal injury, consistent with the intracellular localization of the target antigen (132). This mechanism is most plausible for CNS manifestations such as encephalitis, cerebellar involvement, and myelopathy.

However, recent experimental evidence indicates that CV2/CRMP5 antibodies can directly contribute to painful neuropathy. Patient-derived antibodies bound CRMP5 in dorsal root ganglia and spinal dorsal horn, increased sensory neuron excitability, and induced mechanical hypersensitivity in rats; these effects were prevented by CRMP5 antibody depletion or epitope blockade. A CRMP5 immunization model further reproduced mechanical hypersensitivity, which was alleviated by B-cell depletion (133).

Thus, anti-CV2/CRMP5 disease may involve phenotype-dependent mechanisms: cytotoxic T-cell-mediated injury likely drives irreversible central manifestations, whereas antibody-mediated sensory neuron hyperexcitability may contribute to neuropathic pain. Whether similar direct antibody effects occur in the CNS remains unresolved.

## 9 Treatment

Advances in the understanding of AE immunopathology have directly informed the development of therapeutic strategies. While current treatment remains largely based on broad immunosuppression and antibody removal, increasing knowledge of disease-specific immune mechanisms has stimulated the development of targeted therapies aimed at B cells, T cells, cytokine pathways, and pathogenic antibodies. However, translating these mechanistic insights into effective clinical interventions remains challenging, particularly because of BBB constraints and persistent intrathecal immune responses. Therefore, both the therapeutic rationale and the practical limitations of emerging treatments should be considered. To directly address differences in evidence type, AE subtype relevance, and major limitations across selected targeted therapies, including IL-6 blockade, FcRn inhibition, CAAR-T cells, and proteasome inhibition, these agents are summarized in **Table 2**.

**Table 2:** Therapeutic strategies and their mechanisms of action in autoimmune encephalitis.

Broad therapeutic strategy	Specific target/category	Representative agents/therapies	Detailed mechanism of action	Clinical considerations/stage	Evidence level/key limitations
Conventional Immunotherapy	First-line Immunotherapy	Corticosteroids, IVIG, Plasma Exchange	Broad immunosuppression; neutralization/clearance of pathogenic antibodies in the periphery.	Foundation for acute-phase management.	Level 2: Widely used foundation therapy; response may be incomplete in severe or refractory cases
	Second-line Immunotherapy (Cytotoxic)	Cyclophosphamide	Potent immunosuppression, targeting rapidly dividing immune cells (e.g., B cells, T cells).	Used for severe or refractory cases.	Level 3:Useful in refractory disease; infection and toxicity risks
B-Cell Targeted Therapies	Targeting B-cell Surface Antigens	Rituximab	Depletes CD20^+^ B cells, preventing their differentiation into antibody-producing plasma cells.	Standard second-line therapy for NMDAR, LGI1, CASPR2 encephalitis; does not eliminate mature plasma cells (134).	Level 2:Does not eliminate mature plasma cells; delayed effect possible
		Inebilizumab	Targets CD19, covering a broader spectrum of B-lineage cells (including some plasmablasts).	Approved for NMOSD; potentially more comprehensive for AE, under investigation (135).	Level 3-4:AE-specific evidence remains limited
	Targeting Plasma Cells	Daratumumab	Targets CD38, directly depleting antibody-secreting plasma cells.	Used in refractory cases (e.g., CASPR2 encephalitis); high risk of infections; case-report level evidence (136).	Level 4:Case-report level evidence; infection risk
		Bortezomib	Proteasome inhibitor, induces apoptosis in plasma cells.	Used in refractory cases.	Level 3-4:Limited AE-specific efficacy data; infection, neuropathy, access issues
T-Cell & Cytokine Targeted Therapies	Targeting T-cell Signaling	Tacrolimus	Inhibits calcineurin in the TCR signaling pathway, suppressing T-cell activation and proliferation.	Used as long-term maintenance; may accelerate early recovery; long-term benefit uncertain (137).	Level 3:Long-term toxicity; limited AE-specific controlled data
		Sirolimus (Rapamycin)	Inhibits mTOR pathway, halting T-cell cycle progression; may enhance Treg function.	Limited evidence in AE; efficacy not prominent in available studies (138–140).	Level 4:Limited AE evidence; monitoring required
	Cytokine Blockade	Tocilizumab	Blocks IL-6 signaling, inhibiting B-cell differentiation and plasma cell survival; restores Th17/Treg balance.	Used in refractory NMDAR, LGI1 encephalitis; shows promise as rescue therapy (141, 142).	Level 3:Tocilizumab has observational evidence; infection risk
		JAK inhibitors (e.g., Tofacitinib)	Broadly inhibits JAK/STAT pathway downstream of multiple cytokines, indirectly suppressing immune cell activation.	Anecdotal use in refractory AE; based on efficacy in other autoimmune diseases (143, 144).	Level 4:Evidence mainly from autoimmune diseases AE data limited
	Immune Regulation	Low-dose IL-2	Selectively expands and activates regulatory T cells (Tregs), restoring immune balance.	Early studies show feasibility and rapid effect in refractory AE; requires large-scale trials (145).	Level 4:Early-stage evidence; requires further validation.
Precision-Targeted Therapies	Accelerating Antibody Clearance	FcRn inhibitors (e.g., Rozanolixizumab)	Blocks FcRn, accelerating the degradation of all IgG antibodies, including pathogenic ones.	Rapidly reduces peripheral antibody levels; effect on CNS antibodies requires validation (146, 147).	Level 4:AE-specific evidence not established; may not affect intrathecal antibodies.
	Neutralizing Antibody Effect	IgG-degrading enzymes (IdeS, EndoS)	IdeS: Cleaves IgG, rendering it ineffective. EndoS: Removes IgG glycan, reducing effector functions.	Rapid reduction in antibody levels/toxicity; preclinical stage for AE (148–150).	Level 4:Preclinical or early-stage evidence; toxicity and practicality concerns.
	Stabilizing Synaptic Receptors	Open-channel blockers (e.g., Ketamine)	Stabilizes NMDAR anchoring at the synapse by enhancing interaction with scaffolding proteins, preventing antibody-mediated internalization.	Effective in animal models; suggests novel adjunctive strategy; clinical translation needs research (160).	Level 4:Mostly preclinical; clinical application uncertain.
		Ephrin-B2/EphB2R pathway agonists	Activates EphB2 receptor, promoting clustering of NMDARs at synapses, preventing internalization.	Preclinical stage; current administration methods (e.g., intracerebroventricular) are not clinically practical.	Level 4:Preclinical stage; delivery route and safety remain unresolved.
Advanced Exploratory Therapies	Cellular Therapy	NMDAR-CAAR-T cells	Uses NMDAR antigen as “bait” to specifically eliminate pathogenic autoantibody-producing B cells.	Preclinical stage; high specificity; generalizability and impact on clinical phenotype need validation (151).	Level 4:Preclinical stage; high specificity but clinical safety and feasibility unknown.

Evidence grading: Level 1, evidence from randomized controlled trials or high-quality prospective studies in AE; Level 2, evidence from multicenter cohorts, large observational studies, or widely accepted clinical practice; Level 3, evidence from small retrospective cohorts, case series, or limited subtype-specific studies; Level 4, evidence from case reports, preclinical studies, mechanistic rationale, or extrapolation from other autoimmune diseases.

### 9.1 Conventional treatment

**Figure 5:**
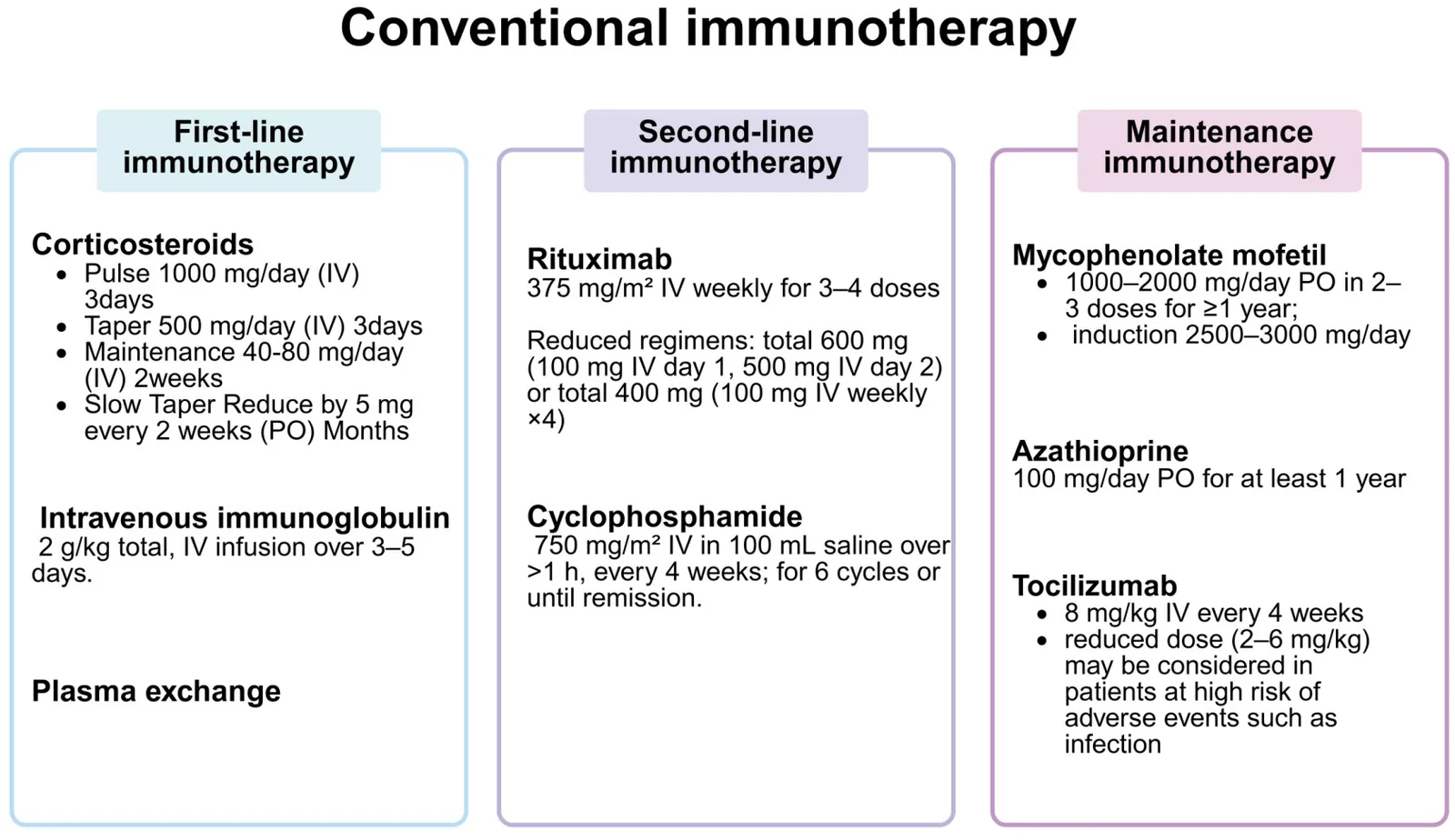
Conventional immunotherapy for AE. Immunotherapy is applied in a stepwise manner: first-line therapy (corticosteroids, IVIG, plasma exchange) for rapid control, second-line therapy (rituximab, cyclophosphamide) for refractory cases, and maintenance therapy (mycophenolate mofetil, azathioprine, tocilizumab) to prevent relapse.

The treatment of AE typically follows an escalation therapy approach (**Figure 5**) (152). First-line treatments include corticosteroids, IVIG, and plasma exchange. If first-line therapy proves ineffective or suboptimal, second-line immunotherapy is initiated, which includes anti-CD20 mAbs such as rituximab and intravenous cyclophosphamide. If there is no significant improvement after intensified first-line immunotherapy (e.g., multiple cycles of IVIG) or following second-line treatment, long-term (maintenance) immunotherapy may be considered. Maintenance immunotherapy regimens include mycophenolate mofetil, azathioprine, and repeated doses of rituximab. As summarized in **Table 3**, several clinical trials are underway to evaluate targeted therapies in autoimmune encephalitis, including CD19 depletion with inebilizumab, IL-6 receptor blockade with satralizumab, proteasome inhibition with bortezomib, sequential CD20/CD38 depletion strategies, and CD20-targeting agents such as ocrelizumab. These trials highlight efforts to expand beyond conventional immunotherapy toward mechanism-based interventions across different AE subtypes and refractory populations. However, the evidence level differs substantially among these targeted therapies, ranging from ongoing clinical trials to case-based clinical experience and preclinical studies. Therefore, their potential application should be interpreted according to evidence type, AE subtype relevance, and key limitations, including infection risk, treatment accessibility, BBB penetration, and persistence of intrathecal antibody production.

**Table 3:** Summary of clinical trials for targeted therapies in autoimmune encephalitis.

Mechanism/target	Drug/biologic	Trial ID	Phase	AE subtype/population	Design (arms)	Primary endpoint (examples)
CD19 (B cell/plasmablast) depletion	Inebilizumab	NCT04372615	Phase 2 (randomized, double-blind, placebo-controlled)	Anti-NMDAR encephalitis; adjunct to first-line therapy	Inebilizumab vs placebo + standard first-line	mRS at 16 weeks; safety
IL-6 receptor blockade	Satralizumab	NCT05503264	Phase 3 (basket)	NMDAR-IgG^+^ or LGI1-IgG^+^ AIE	Randomized, double-blind, placebo-controlled; multicenter	Efficacy & safety; PK/PD over 52-week DB period
Proteasome inhibition (long-lived plasma cell depletion)	Bortezomib	NCT03993262	Phase 2	Severe/refractory autoimmune encephalitis	Interventional; details per registry	Efficacy and safety endpoints
Sequential CD20 (B cell) → CD38 (plasma cell) depletion	Ofatumumab → Daratumumab	NCT06867991	Phase 3	Severe anti-NMDAR autoimmune encephalitis	Sequential ofatumumab then daratumumab	Efficacy and safety
CD20 B-cell depletion	Ocrelizumab	NCT03835728	Phase 2 (pilot RCT)	Autoimmune encephalitis (new diagnosis)	Randomized, double-blind, placebo-controlled	Efficacy and safety (~6 months)

### 9.2 B cell-targeted immunotherapy

In the treatment of AE, mAbs have gradually emerged as important therapeutic strategies, primarily targeting B cells, plasma cells, or inflammatory cytokines. Rituximab eliminates CD20^+^ B cells, thereby preventing their differentiation into plasma cells and reducing pathogenic antibody production. It is commonly used as a second-line therapy for NMDAR-, LGI1-, and CASPR2-antibody-associated encephalitis, with its use in AE supported mainly by non-randomized clinical experience and observational evidence summarized in previous studies (134). However, rituximab cannot deplete mature CD138^+^ plasma cells, and residual antibodies may persist in some patients, especially when antibody production is maintained by plasmablasts, long-lived plasma cells, or intrathecal immune responses. Inebilizumab, targeting CD19, covers a broader spectrum of B cell lineages, including subsets of plasmablasts and plasma cells, and may achieve more complete depletion compared with rituximab. It has been approved for AQP4^+^ neuromyelitis optica spectrum disorder (NMOSD) (135) and is theoretically applicable to antibody-mediated AE, with ongoing studies under investigation. At present, AE-specific evidence for inebilizumab is mainly trial-stage evidence, particularly in anti-NMDAR encephalitis, rather than established routine clinical evidence. Nevertheless, AE-specific efficacy data remain limited, and key concerns include infection, hypogammaglobulinemia, treatment accessibility, and uncertain effects on CNS-compartmentalized antibody production. Daratumumab, an anti-CD38 antibody, reduces antibody secretion by directly depleting plasma cells. Case reports have described its use in refractory anti-CASPR2 encephalitis, where short-term antibody reduction was achieved; however, its application was associated with a considerable risk of severe infections (136).

Proteasome inhibition with bortezomib represents another plasma cell-directed strategy. By targeting antibody-secreting plasma cells, bortezomib may be particularly relevant to severe or refractory antibody-mediated AE with persistent antibody production, especially anti-NMDAR encephalitis. However, current AE evidence is derived mainly from case reports, small case series, and retrospective Class IV evidence rather than randomized controlled trials. Therefore, bortezomib should be regarded as an investigational rescue therapy rather than an established standard treatment. Key limitations include infection risk, cytopenia, peripheral neuropathy, limited treatment accessibility, uncertain BBB penetration, and incomplete efficacy against long-lived intrathecal plasma cells or CNS-resident antibody-secreting cells.

IL-6 plays a pivotal role in B cell differentiation into plasma cells, antibody production, and survival, as well as in promoting Th17 cell differentiation and driving inflammation (153). Accordingly, blockade of the IL-6/IL-6R signaling pathway can suppress immune responses at multiple levels, thereby reducing autoantibody levels. Agents such as tocilizumab, satralizumab, and siltuximab inhibit B cell differentiation and plasma cell survival by disrupting IL-6 signaling. However, their evidence levels in AE differ substantially. Tocilizumab has been used mainly as rescue therapy in refractory AE, particularly anti-NMDAR and anti-LGI1 encephalitis, with support primarily from case reports, small case series, and retrospective observational studies rather than randomized controlled trials. Satralizumab is currently being evaluated in a phase III randomized, double-blind, placebo-controlled trial involving anti-LGI1 encephalitis and anti-NMDAR encephalitis, and therefore remains a trial-stage therapy in AE. Evidence for siltuximab in AE is more limited and is mainly based on mechanistic rationale or extrapolation from other inflammatory diseases. These therapies have shown efficacy in refractory NMDAR encephalitis, though large-scale clinical trials are still needed to confirm their long-term efficacy and safety. Important limitations of IL-6/IL-6R blockade include infection risk, treatment accessibility, and uncertain effects on BBB-compartmentalized inflammation, intrathecal antibody production, and CNS-resident immune responses.

Current therapeutic approaches such as corticosteroids, rituximab, and plasma exchange rely on broad-spectrum immunosuppression or nonspecific antibody clearance, which may result in severe complications such as sepsis and thrombosis (134). Moreover, they are unable to selectively eliminate pathogenic B cell populations. Chimeric antigen receptor T cell (CAR-T) therapy, an individualized cellular immunotherapy initially developed for hematologic malignancies (154), is now expanding to neurological disorders, including primary brain tumors and autoimmune neurological diseases (155). Recently, Reincke and colleagues developed NMDAR-specific chimeric autoantibody receptor T cells (NMDAR-CAAR T cells), which utilize the self-antigen as a “bait” to engage B cell receptors, thereby selectively eliminating autoreactive B cells that produce pathogenic antibodies and potentially reducing disease relapse and long-term adverse effects (151). This strategy is currently supported by preclinical evidence and is most directly applicable to anti-NMDAR encephalitis, rather than to AE subtypes more broadly. Nevertheless, the animal models used in this study were generated through passive transfer of autoreactive B cells and do not fully recapitulate the complex clinical phenotype of anti-NMDAR encephalitis, making it difficult to evaluate comprehensive effects on neuronal injury or behavioral changes. Additionally, some rare antibody epitopes are not covered by current CAAR designs, and thus the generalizability of this strategy requires validation in larger patient cohorts. Key limitations include individualized manufacturing, high cost, treatment accessibility, potential cellular therapy-related toxicity, uncertain CNS trafficking across the BBB, and unclear efficacy against established intrathecal antibody production.

### 9.3 T cell-targeted immunotherapy

Calcineurin inhibitors (CNIs), such as cyclosporine and tacrolimus, act by inhibiting calcineurin within the T-cell receptor (TCR) signaling pathway, thereby blocking IL-2 production, suppressing T-cell activation and proliferation, and indirectly reducing antibody generation and inflammatory responses. In AE, observational evidence suggests that tacrolimus, when used as a long-term immunotherapy, may accelerate early recovery. However, its benefits for long-term prognosis and relapse prevention remain uncertain (137).

mTOR inhibitors, represented by sirolimus and everolimus, primarily exert their effects by blocking the mTOR pathway, preventing T cells from progressing from the G1 to S phase, thereby reducing effector T-cell activation. At the same time, they may enhance the proportion of regulatory T cells (Tregs), which theoretically could ameliorate T cell-driven neuroinflammation. These agents have been applied in certain autoimmune diseases with predominant T-cell involvement (139, 140). In AE, evidence is scarce; a prospective open-label study in anti-Hu paraneoplastic neurological syndromes (PNS) reported improvement in a few individual cases, although the overall efficacy was limited (138).

Low-dose IL-2 therapy selectively expands Tregs and suppresses aberrantly activated effector T cells. Multiple clinical studies and early trials in systemic lupus erythematosus, dermatomyositis, and myasthenia gravis have demonstrated that low-dose IL-2 can effectively expand Tregs and improve disease activity (156, 157). Exploratory research in CNS autoimmune disorders, such as multiple sclerosis, has also indicated potential benefit (158). Jung-Ah Lim and colleagues evaluated the feasibility and safety of low-dose IL-2 in refractory AE patients, reporting that some patients experienced improvement after just 1 to 2 treatment cycles, suggesting a rapid onset of immunomodulatory effects (145). Nevertheless, the application of low-dose IL-2 in AE remains largely theoretical and exploratory, with no large-scale clinical trials or systematic studies to date confirming its efficacy.

In terms of cytokine and chemokine pathway blockade, IL-6 has been identified as a key mediator in AE, particularly in promoting Th17 differentiation and impairing Treg function. Tocilizumab, an IL-6/IL-6R pathway inhibitor, has been used in select cases of refractory anti-NMDAR and anti-LGI1 encephalitis where conventional therapy failed, showing clinical improvement (141, 142, 152). This highlights its potential as a rescue treatment, though large-scale validation remains lacking. Janus kinase (JAK) inhibitors, such as tofacitinib and ruxolitinib, broadly block cytokine-mediated JAK/STAT signaling and thereby indirectly inhibit T-cell activation. They have demonstrated efficacy in other autoimmune diseases (143) and have been trialed in isolated cases of refractory AE (144). While the current evidence is limited, these agents offer novel therapeutic avenues for treatment-resistant AE.

### 9.4 Precision-targeted therapies

The neonatal Fc receptor (FcRn) maintains IgG recycling and homeostasis. FcRn inhibitors such as rozanolixizumab block FcRn, enhance IgG catabolism, and rapidly lower pathogenic IgG; in myasthenia gravis, rozanolixizumab reduces acetylcholine receptor antibody titers, improves muscle weakness, and has advanced to phase III trials (147), with similar agents under evaluation in neuromyelitis optica spectrum disorder (NMOSD) (146). These findings support FcRn inhibition as a rational strategy in AE. However, AE-specific evidence for FcRn inhibition remains limited and is currently strongest as mechanistic rationale or early/trial-stage clinical evaluation rather than established efficacy. This strategy is most relevant to surface antibody-mediated AE subtypes in which pathogenic IgG plays a central role, such as anti-LGI1, anti-NMDAR, anti-CASPR2, and anti-GABA_B_R encephalitis. Nonetheless, because current FcRn inhibitors act mainly on peripheral IgG and do not target intrathecal antibody production, their capacity to meaningfully reduce CNS antibody burden is unclear and could ultimately constrain their clinical benefit in AE. Additional concerns include infection risk related to systemic IgG reduction, treatment accessibility, limited or uncertain BBB penetration, and the possibility that CNS-resident antibody-secreting cells may continue to sustain disease despite peripheral IgG lowering.

IgG-degrading enzymes offer a potential strategy for selectively reducing pathogenic antibodies. IdeS, clinically developed as imlifidase, cleaves human IgG at the lower hinge region to generate F(ab’)2 and Fc fragments, thereby rapidly impairing Fc-mediated effector functions. Current clinical evidence mainly comes from HLA-sensitized kidney transplant candidates and anti-glomerular basement membrane disease (149), while its use in hematologic disorders remains exploratory rather than clinically established (150). Therefore, IdeS provides an important proof-of-concept for rapid IgG neutralization, but its applicability to autoimmune encephalitis requires further validation. EndoS removes the conserved N-linked glycan from the IgG Fc region, reducing IgG binding to C1q and Fcγ receptors and consequently weakening complement activation and antibody-dependent cytotoxicity. Although EndoS has shown efficacy in murine autoimmune models (148), its application in autoimmune encephalitis is still limited to preclinical research. In our view, IgG-degrading or IgG-modifying enzymes are promising for antibody-mediated autoimmune encephalitis because of their rapid and direct effects on pathogenic IgG; however, concerns regarding antibody rebound, immunogenicity, infection risk, BBB penetration, and disease heterogeneity must be addressed before clinical translation.

Identification of the N368-G369 region on GluN1 as a core pathogenic epitope of anti-GluN1 antibodies has enabled the development of epitope-focused “occupancy blockade” strategies. Here, occupancy blockade means that a non-pathogenic antibody-like molecule occupies or shields a disease-relevant epitope on GluN1, thereby preventing pathogenic anti-GluN1 antibodies from binding to the same or an overlapping site. The humanized monovalent IgG ART5803 binds with high affinity to the GluN1 N-terminal domain at a conformational epitope overlapping the N368-G369 region. Through this epitope-shielding mechanism, ART5803 blocks pathogenic antibody binding, prevents antibody-induced NMDAR internalization, and reverses behavioral deficits in a marmoset model (159). ART5803 is therefore better understood as an epitope-protective molecule rather than a soluble decoy, and it is currently being developed as a potential occupancy-blockade therapy for anti-NMDAR encephalitis and related autoimmune neuropsychiatric disorders.

Therapies targeting antibody-mediated NMDAR dysfunction increasingly aim to preserve synaptic receptor stability. Open-channel blockers (OCBs) such as ketamine and MK-801 counteract antibody-induced changes in NMDAR membrane dynamics (160). By binding within the channel pore, OCBs restrict lateral diffusion, prolong synaptic residency, and promote conformational changes that enhance interactions with PDZ-domain scaffolding proteins such as PSD-95, thereby maintaining synaptic anchoring. In animal models, ketamine prevents receptor loss and ameliorates behavioral abnormalities including anxiety, anhedonia, and sensorimotor-gating deficits. Ephrin-B2-based interventions act through a complementary mechanism: EphB2 activation enhances synaptic NMDAR clustering, preserves surface expression, and reduces antibody-mediated internalization, improving learning, memory, and synaptic plasticity in preclinical studies. Nonetheless, the need for continuous intracerebroventricular delivery and the incomplete reversal of established disease currently limit translational applicability.

Collectively, FcRn inhibition, IgG-modifying enzymes, epitope-targeted occupancy blockade, and synapse-stabilizing strategies (OCBs, ephrin-B2) illustrate a shift toward precision interventions that directly modulate pathogenic IgG or NMDAR synaptic stability. Future work should prioritize clinically feasible agents capable of safely enhancing receptor retention and function within the CNS, ideally with noninvasive delivery and robust intrathecal efficacy.

Despite the promise of these emerging therapies, a major translational challenge in autoimmune encephalitis is the BBB. Many targeted therapies, including monoclonal antibodies, FcRn inhibitors, and cellular therapies, primarily act in the peripheral immune compartment and may have limited access to the central nervous system. Although BBB disruption occurs during active disease, the extent and duration of this permeability vary considerably among patients and disease stages. Moreover, persistent intrathecal antibody production and CNS-resident immune populations may continue to drive pathology even when peripheral immune responses are effectively suppressed. Therefore, future therapeutic development should focus not only on selective immune modulation but also on strategies capable of achieving sustained effects within the CNS compartment.

## 10 Conclusion and future perspectives

AE arises from the interplay of antibody-mediated synaptic dysfunction, immune-cell-driven inflammatory cascades, and BBB disruption, which together disturb neural circuits and drive clinical manifestations. In anti-NMDAR encephalitis, pathogenic antibodies not only induce receptor internalization but also alter membrane dynamics and extrasynaptic signaling, while IgG4 antibodies in anti-LGI1 and anti-CASPR2 encephalitis primarily cause “functional silencing” by disrupting protein-protein interactions without complement activation. Paraneoplastic mechanisms play a central role in anti-GABA_B_R encephalitis, whereas encephalitides associated with intracellular neuronal antigens are dominated by cytotoxic T-cell-mediated irreversible neuronal injury and generally show poor response to immunotherapy. Recent evidence highlights the contribution of cytokine networks, B- and T-cell subsets, microglial activation, and gut-brain axis interactions, underscoring that AE is not a purely antibody-driven disorder but rather a complex immune-neural pathology (**Table 4**). Moving forward, advances in mechanistic subtyping, the discovery of multimodal biomarkers, and the development of targeted immunotherapies-including CNS-penetrant B- and plasma-cell-directed agents, IL-6/JAK-STAT blockade, FcRn inhibitors, IgG-modifying enzymes, antigen-specific decoys, and synaptic stabilizers—offer opportunities to move beyond broad immunosuppression (**Table 2**). Future clinical trials should integrate mechanistic readouts, stratify patients by antibody subtype, genetic background, and cytokine profile, and incorporate systematic tumor screening where relevant. Together, these efforts will enable the transition from empirical therapy to mechanism-based, subtype-specific precision medicine in AE.

**Table 4:** Comparative overview of antibody characteristics in autoimmune encephalitis subtypes.

AE subtype	Antibody class	Antigen	Antigen location	Functional consequence/evidence grade	Main clinical manifestations	Tumor association/screening implication
Anti-NMDAR Encephalitis	IgG1	NMDAR (GluN1 subunit)	Postsynaptic membrane, extracellular	Antibody cross-links and internalizes NMDAR, leading to decreased receptor density and impaired synaptic transmission Evidence: Level 1	Psychiatric symptoms, seizures, movement disorders, autonomic dysfunction	Ovarian teratoma common; pelvic imaging, especially in young women.
Anti-LGI1 Encephalitis	IgG4	LGI1 (binds ADAM22/23)	Synaptic cleft, secreted protein	Disrupts LGI1-ADAM22/23 complex, leading to reduced AMPAR function Evidence: Level 1-2	Memory impairment, seizures (faciobrachial dystonic seizures), cognitive impairment	Usually non-paraneoplastic; individualized screening.
Anti-CASPR2 Encephalitis	IgG4	CASPR2 (associated with Kv1 channel complex)	Axonal juxtaparanodal region, presynaptic membrane	Disrupts Kv1 channel localization, leading to neuronal hyperexcitability Evidence: Level 2	Peripheral nerve hyperexcitability, Morvan’s syndrome, limbic encephalitis	Associated with thymoma; screen in Morvan’s/PNS hyperexcitability.
Anti- GABA_B_R Encephalitis	IgG1	GABA_B_ receptor (B1 subunit)	Postsynaptic membrane, extracellular	Blocks inhibitory GABAergic signaling, leading to neuronal hyperexcitability Evidence: Level 2	Refractory seizures, psychiatric/behavioral abnormalities	Strongly linked to SCLC; chest CT/PET-CT recommended.
Anti-DPPX Encephalitis	IgG1	DPPX (regulatory subunit of Kv4.2 channels)	Extrasynaptic membrane protein	Likely reduces A-type K^+^ current, leading to neuronal hyperexcitability Evidence: Level 1-2	Seizures, cognitive impairment, diarrhea, weight loss	Tumors rare; lymphoma reported. Screen if systemic symptoms.
Anti-GluRδ2 Encephalitis	IgG	GluRδ2 (Glutamate receptor δ-2 subunit)	Cerebellar granular layer postsynaptic membrane	Interferes with glutamate signaling and synaptic plasticity Evidence: Level 3-4	Cerebellar ataxia, cases are rare	Tumor link unclear; age-/phenotype-based screening.
Anti-Hu Encephalitis (ANNA-1)	IgG	Hu protein (ELAVL family RNA-binding protein)	Neuronal nucleus (intracellular antigen)	Predominantly cytotoxic T-cell-mediated neuronal injury; direct antibody pathogenicity remains uncertain. Evidence: Level 1–2 for T-cell injury; Level 3–4 for antibody pathogenicity.	Limbic encephalitis, sensory neuronopathy, associated with small cell lung cancer	High-risk paraneoplastic; mainly SCLC. Systemic search needed.
Anti-Ri Encephalitis (ANNA-2)	IgG	Nova1/2 protein (RNA-binding protein)	Neuronal nucleus (intracellular antigen)	T cell-mediated neuronal damage Evidence: Level 2	Opsoclonus-myoclonus-ataxia, eye movement disorders, associated with breast cancer/small cell lung cancer	Linked to breast, SCLC, and gynecologic tumors.
Anti-CV2 encephalitis (CRMP5-related)	IgG	CRMP5	Intracellular/cytoplasmic antigen in neurons and oligodendrocytes	T cell-mediated demyelination and neuronal damage Evidence: Level 2	Limbic encephalitis, myelitis, optic neuritis, associated with thymoma/small cell lung cancer	Strong paraneoplastic link; SCLC/thymoma, chest imaging.
Anti-Yo Encephalitis (PCA-1)	IgG	Yo antigen (CDR2/CDR2L)	Cerebellar Purkinje cell cytoplasm	Cytotoxic T cell-mediated Purkinje cell loss Evidence: Level 1-2	Cerebellar ataxia, associated with breast cancer/ovarian cancer	Linked to breast/ovarian cancers; breast and pelvic screening.

Tumor associations are summarized to highlight clinically relevant screening implications and do not indicate that all cases are paraneoplastic. Screening strategies should be individualized according to antibody type, age, sex, smoking history, clinical phenotype, and local guidelines.

Evidence grading: Level 1 = direct functional/pathological evidence; Level 2 = probable evidence from consistent experimental or cohort data; Level 3 = associative evidence without causal validation; Level 4 = exploratory or case-level evidence.
